# Effects of Diet Control and Telemedicine-Based Resistance Exercise Intervention on Patients with Obesity and Knee Osteoarthritis: A Randomized Control Trial

**DOI:** 10.3390/ijerph18157744

**Published:** 2021-07-21

**Authors:** Yen-I Hsu, Ying-Chou Chen, Chia-Lun Lee, Nai-Jen Chang

**Affiliations:** 1 Department of Sports Medicine, Kaohsiung Medical University, Kaohsiung 807, Taiwan; katekate411@gmail.com; 2 Nutritional Therapy, Department of Internal Medicine, Kaohsiung Chang Gung Memorial Hospital, Chang Gung University College of Medicine, Kaohsiung 833, Taiwan; 3 Division of Rheumatology, Allergy, and Immunology, Department of Internal Medicine, Kaohsiung Chang Gung Memorial Hospital, Chang-Gung University College of Medicine, Kaohsiung 833, Taiwan; slechen1939@gmail.com; 4 Center for Physical and Health Education, National Sun Yat-Sen University, Kaohsiung 804, Taiwan; karenlee1129@gmail.com; 5 Ph.D. Program in Biomedical Engineering, College of Medicine, Kaohsiung Medical University, Kaohsiung 807, Taiwan; 6 Regenerative Medicine and Cell Therapy Research Center, Kaohsiung Medical University, Kaohsiung 807, Taiwan; 7 Department of Medical Research, Kaohsiung Medical University Hospital, Kaohsiung 807, Taiwan

**Keywords:** exercise, body composition, cartilage, telemedicine, healthcare, nutrition

## Abstract

This study investigated the effects of home-based nutritional and telemedicine-based resistance exercise interventions on improving body composition, blood biochemistry, and lower-limb functional performance. In total, 66 obese patients with mild-to-moderate knee osteoarthritis were randomly divided into a diet control group (D), elastic band resistance exercise group (E), and diet control plus elastic band exercise group (D + E). Each group was supervised by a clinical dietitian and follow-up was conducted via telephone calls or a communication application to track the participants’ progress. After 12 weeks of intervention, the D (*p* < 0.001) and D + E (*p* < 0.001) groups achieved significant weight loss. The D + E group exhibited a significant reduction in body fat relative to the D (*p* = 0.019) and E (*p* = 0.012) groups. Compared with the D (*p* = 0.002) and E (*p* = 0.019) groups, the D + E group achieved significant improvements in the timed up-and-go test and Western Ontario and McMaster Universities Osteoarthritis total scale. The D + E group experienced significant improvements in total cholesterol (*p* = 0.001), low-density lipoprotein cholesterol (*p* = 0.01), and triglyceride levels (*p* = 0.007) relative to other groups. In conclusion, individual diet control intervention combined with telemedicine-based resistance exercise intervention significantly improved the body composition, blood biochemistry, and lower-limb functional performance of the investigated population with comorbid conditions.

## 1. Introduction

Obesity is a key public health issue. It promotes the development of osteoarthritis (OA), which causes joint stiffness, pain, and declining functional performance [1,2]. In addition, patients with obesity usually lack access to nutritional information and do not know how to manage their nutritional intake to implement a healthy diet [3,4]. European guidelines for obesity management recommend weight reduction as the nonpharmacological treatment for this population [5]. A previous study suggested that a 5% body weight reduction produced improved clinical symptoms including pain relief and functional mobility improvement [6]. However, adverse effects were observed when sedentary patients with obesity and knee OA were subjected to a very low-energy diet; specifically, they developed notable bad breath, intolerance to cold, flatulence, and loss of lean body mass [7].

The American College of Sports Medicine proposed the position that “exercise is medicine”. Exercise, which includes aerobic and resistance training, is an interventional recommendation for treating OA [8,9]. Patients with knee OA who underwent an aerobic exercise intervention reported significantly reduced joint pain and increased joint range of motion [10]. However, obesity increases knee joint load due to the heavy weight borne by the knees. Therefore, patients with obesity and comorbid knee OA may experience increased joint pain during aerobic exercise due to the compressive impact force on the knee joints, leading to low adherence [11,12]. For strength training, resistance-band training serves as a relatively cheaper and more convenient alternative to machine-based resistance training for patients with knee OA [13,14] and patients with sarcopenic obesity [15]. However, the clinical benefits of these therapeutic exercise programs decline over time, which is likely due to poor adherence [16]. Engagement in therapeutic exercise among people in osteoarthritic populations is influenced by a complex interplay between physical, personal, and social–environmental factors [17]. These barriers to performing therapeutic exercises include decreased motivation (e.g., patients may only complete exercises when provided with action coping plans and/or audio/video exercises), pain and physical limitations, nonpositive therapeutic exercise information, and lack of professional support [18]. In addition, the limitations of elastic-band training include difficult to load control and quantify the specific an amount of resistance [19]. Therefore, increasing adherence among the aforementioned population to therapeutic exercise which can be feasibly performed as graded exercise at home through the provision of instruction is a key strategy [20].

Telemedicine is a promising strategy, particularly for the early stages of a home-based therapeutic exercise program; it allows for exercise techniques to be corrected for safety, and an exercise program can be adjusted to suit an individual’s physical ability and goals [21,22,23]. In our previous study, a 12-week telemedicine-based elastic band intervention was implemented with regular follow-up via phone calls or communication software by the clinical staff of a medical center; the intervention led to improved muscle strength, dynamic balance, and physical function in patients with comorbid type 2 diabetes mellitus and knee OA [24]. Collectively, these findings indicate that telemedicine-based elastic band exercise is safe for patients with various conditions. In addition, systematic reviews and randomized controlled trials examining people with knee and hip OA have highlighted that e-health technologies such as telehealth and mobile health may provide more opportunities for interactions with exercise professionals and increase the exercise adherence of patients, particularly in the OA population [17].

Previous studies have examined patients with obesity and knee OA and compared the effects of weight loss only [6,25], exercise only [26,27], and weight loss + exercise [12,28,29,30]. Based on aforementioned studies, the literature has not examined the feasibility of achieving weight loss, improving blood biochemical values, and enhancing functional performance through the provision of nutritional advice and elastic band resistance exercise instructions provided by a dietitian combined with implementation of follow-up using telemedicine to track participants’ progress, particularly with respect to the aforementioned population with comorbid obesity and knee OA. Therefore, this study investigated the effects of an individualized nutritional and elastic band resistance exercise intervention program delivered by a dietitian who specializes in sports medicine through telemedicine and applied to older patients with obesity and knee OA. Regarding functional assessments, the Western Ontario and McMaster Universities Osteoarthritis scale (WOMAC) scale is a high intraclass correlation coefficient self-questionnaire used to assess the health status of patients with knee OA [24,31]. The Osteoarthritis Research Society International recommends the timed up-and-go (TUG) test to assess the dynamic balance of patients with knee or hip OA [32]. The TUG test is highly reliable for patients with mild-to-moderate knee OA [24,33].Therefore, the primary outcome was assessed by reviewing the patients’ body composition and WOMAC scores. The secondary outcomes were assessed by reviewing the patients’ blood biochemical analysis and TUG test results.

## 2. Materials and Methods

### 2.1. Study Design and Setting

This research was approved by the Institutional Review Board of the Chang Gung Medical Foundation (Approval No. 201800607B0) and conducted in line with current legislation and the Declaration of Helsinki [34]. The protocol of this study was registered with ClinicalTrials.gov (accessed on 20 July 2021) (NCT03973463). This study was a prospective, single-center, randomized control trial. Patients with accessible electronic medical records from visits to Kaohsiung Chang Gung Memorial Hospital in Taiwan were enrolled. A clinical dietitian contacted the patients who met the selection criteria. Patients who had obesity and knee OA and met the criteria of this study were enrolled from the Division of Endocrinology and Metabolism of Kaohsiung Chang Gung Memorial Hospital. After explaining the study purpose to eligible patients and obtaining their written informed consent, we randomly assigned them to either the diet control (D) group, elastic band resistance exercise intervention (E) group, or combined intervention (D + E) group. Random allocations were concealed by using opaque, sealed envelopes (A lot: D group; B lot: E; C lot: D + E group) that were prepared by clinical staff who were not involved in the enrolment process. All patients drew one lot by themselves. Patient identification was recorded on each lot. All enrolled patients in all groups were blinded to their grouping. All outcome measures were assessed by a single investigator from the Division of Endocrinology and Metabolism. The outcomes were assessed before and after the 12-week intervention.

### 2.2. Participants

Patients who were older than 55 years and had a body mass index (BMI) of 27–35 kg/m^2^ were included in the study. In the present study, obesity was defined per the definition established by the National Health Agency; knee OA was diagnosed when X-ray findings indicated a Kellgren and Lawrence (K&L) grade ≤3 [35] and visual analog scale ≥4 out of 10 [36]. The exclusion criteria were as follows: inability to live independently; K&L grade >3; history of hip or knee replacement surgery; history of myocardial infarction; pregnancy or lactation; end-stage liver disease and nephropathy; severe heart disease; lung disease; inability to undergo physical function testing due to conditions such as unstable angina, myocardial infarction, heart failure, severe heart rhythm disorder or second- or third-degree heart conduction block, cardiac aneurysm or aortic aneurysm, or myocarditis or pericarditis; chronic obstructive pulmonary disease accompanied by pulmonary heart disease, untreated or unstable asthma, severe pulmonary hypertension, or pulmonary embolism; and malignant hypertension. Throughout the study, all participants’ drug regimens remained unchanged. This study was conducted at Kaohsiung Chang Gung Memorial Hospital.

### 2.3. Interventions

#### 2.3.1. Diet Control Group

A clinical dietician provided support to the D group and designed an individualized nutritional plan for each participant. These participants also received dietary advice (from the clinical dietitian), health education, and manuals and handouts (covering weight loss and diet) during their first visit to the medical center. Each participant was asked by the clinical dietician to follow a balanced low-energy diet of 1200 kcal/day [7] and update his or her diet record sheet at least three times a week (at least twice and once on weekdays and weekends, respectively). The clinical dietitian followed up with and advised the participants through active phone calls or a communication application (e.g., LINE, FaceTime) once a week for 12 weeks. While performing active calls or mobile application, patient’s interventions were actively instructed by the clinical dietitian based on the individual’s nutritional needs and preferences of each participant.

#### 2.3.2. Elastic Band Resistance Exercise Group

**Figure 1:**
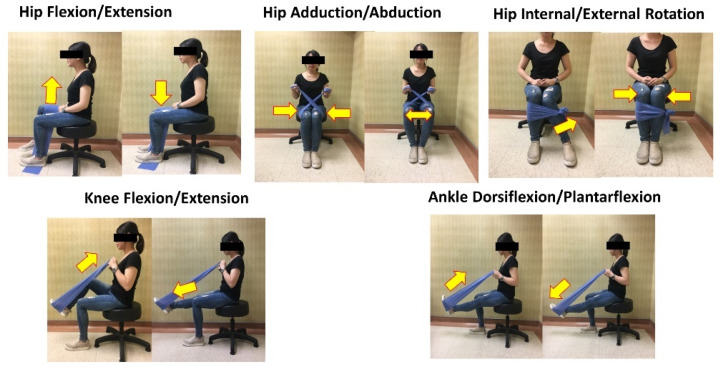
Elastic band resistance exercise intervention.

The elastic band resistance exercise intervention was implemented with the protocol used in our previous study [24]. Due to safety concerns, seated, open-chain exercises were incorporated to strengthen the major muscle groups of the lower extremities. The exercise regime included hip joint extension/flexion, abduction/adduction, external/internal rotation, knee joint extension/flexion, and ankle joint plantarflexion/dorsiflexion movements (Figure 1). Each participant performed 10 repetitions/set of five sets/day of the aforementioned exercise movements 3 days a week for 12 weeks [24]. Exercise intensity was increased by applying more force to the band to provide greater resistance or by switching to a thicker resistance band that created more resistance and thus increased exercise difficulty. A repetition maximum of 10 and rated perceived exertion (RPE) of 13 (range of 6–20) were applied as the standards for the exercise program [13]. As long as participants completed 10 RM and below an RPE of 13, they were prescribed increased exercise difficulty while clinical staff performed telemedicine-based instruction. To ensure that the patients understood the resistance exercise program, each movement was taught by clinical staff at the medical center during the patients’ first visit. The clinical staff specialize in sports medicine and are well trained to prescribe an exercise program. A low-resistance elastic band was used under supervision by telemedicine for familiarization in the first 2 weeks, and bands with progressively higher resistance were used in the subsequent weeks. Thereafter, participants’ compliance with the exercise program was tracked and instruction provided by clinical staff once every week through active phone calls or a communication application (e.g., LINE, FaceTime) for 12 weeks. In addition, they were provided brochures with highlighted notes that served as reminders.

#### 2.3.3. Combined Group

Both the diet control and elastic band resistance program interventions (i.e., the aforementioned protocols) were applied to this group.

### 2.4. Outcome Measures

The clinical staff who collected the data were properly trained in data collection procedures and the execution of follow-up through active phone calls or a communication application (e.g., LINE). The dietitian, who specializes in sports medicine, was responsible for assessing the primary and secondary outcomes before and after the 12-week intervention. In addition, the patients’ blood parameters were monitored by the Department of Laboratory Medicine of Kaohsiung Chang Gung Memorial Hospital.

#### 2.4.1. Primary Outcomes

Body composition results and WOMAC scores were the primary outcomes in the present study. Body composition was determined by performing bioelectrical impedance analysis (BIA) to estimate a patient’s body weight, BMI, body fat percentage, body fat mass, soft lean mass, and lower-limb muscle mass; the analysis was performed using the Body Composition Analyzer (Model IOI 353, Jawon Medical, Seoul, South Korea) [37]. This device has a strong linear correlation (r^2^ = 0.894) and strong agreement (ICC = 0.917) compared with dual-energy X-ray absorptiometry (DXA); Bland–Altman analysis showed that the limit of agreement (%) of this device was 89.3% [38]. Participants were prohibited from engaging in vigorous exercise, consuming food, using a sauna, or drinking excessive amounts of water during the 4 h prior to measurement. To ensure good conductivity, the patients were asked to remove their socks and clean their feet and hands before placing them on the electrode plates for collection of measurements; the related procedures were performed as described in the manufacturer’s operations manual. Body height was measured using automatic height-measuring equipment (HW-3070, Hocom, Taipei, Taiwan). WOMAC items are answered on a five-point Likert scale ranging from 0 (no disability) to 4 (extremely severe disability). A higher score indicates more severe disability. Three subscales for pain, stiffness, and physical function were used, and their scores were added to obtain an overall score. A minimal clinically important difference (MCID) was recognized as a reduction (relative to the baseline score) of at least 14% for the overall score [39], 22.9% for the pain subscale score, 14.4% for the stiffness subscale score, and 19% for the physical function subscale score [40,41].

#### 2.4.2. Secondary Outcomes

Blood biochemical analysis and TUG test results were the secondary outcomes in the present study. The blood biochemical analysis comprised measurements of total cholesterol (normal range <200 mg/dL), low-density lipoprotein cholesterol (LDL; normal range <150 mg/dL), and triglyceride (normal range <150 mg/dL) levels. All blood biochemical analyses were performed by the Department of Laboratory Medicine of Kaohsiung Chang Gung Memorial Hospital. The enzymatic method was used; specifically, a coloring agent was added to a quantitative serum that was then placed in a 37 °C water bath; its absorbance was measured at a wavelength of 505 nm, and the concentration of blood in the serum was calculated. Data were obtained from the electronic medical record system of the medical center.

The TUG test is performed as follows: Upon hearing the “Start” command verbally given by the investigator, the participant stands up from a seated position, walks to a target placed 2.44 m in front of them, turns around, walks back to the chair, and sits down as quickly as possible. The time required to complete this task was measured in seconds. An MCID was recognized as a decrease (relative to baseline) of at least 0.8 s in the total time required to complete the task [42].

### 2.5. Statistical Analyses

The power estimation indicated that a sample size of at least 20 participants in each group was required. The estimations were based on an alpha level of 0.05 and a desired power of 80% with an anticipated dropout rate of 15% [43].

SPSS 25.0 (SPSS, Chicago, IL, USA) was used for data analysis. Data are presented as means and standard deviations (SD). The Shapiro–Wilk test indicated a normal distribution (*p* > 0.05) and the basic data of the participants were analyzed using descriptive statistics, chi-squared tests, and Fisher’s exact test. A paired *t* test was used to analyze the intragroup differences in body composition, WOMAC scale scores, blood biochemical values, and TUG test scores. Analysis of covariance (ANCOVA) was performed, with the covariate being set using pretest data and the groups being independent variables. To address concerns related to the multiple comparison test, the method for controlling type I error was applied in each corresponding multiple comparison method [44]. Post hoc analysis was performed using the Bonferroni method. If a group exhibited nonhomogeneity, an analysis of variance (ANOVA) was performed to test the homogeneity; if the same value (*p* > 0.05) was obtained, then ANCOVA was used to analyze the differences between groups; if homogeneity was not observed (*p* < 0.05), the Welch test was performed to determine the changes between groups and a pairwise comparison was performed using Dunnett’s T3 test instead. The changes from baseline to post-intervention were analyzed using one-way ANOVA, and post hoc testing was performed using Tukey’s range test. The effect size (Cohen’s d), which is the difference between pretest and post-test means divided by their common SD, was calculated and interpreted as small (d = 0.2), medium (d = 0.5), or large (d = 0.8), to show the magnitude of the effect [45]. Statistical significance was established when *p* < 0.05.

## 3. Results

### 3.1. Study Flow for Participants

**Figure 2:**
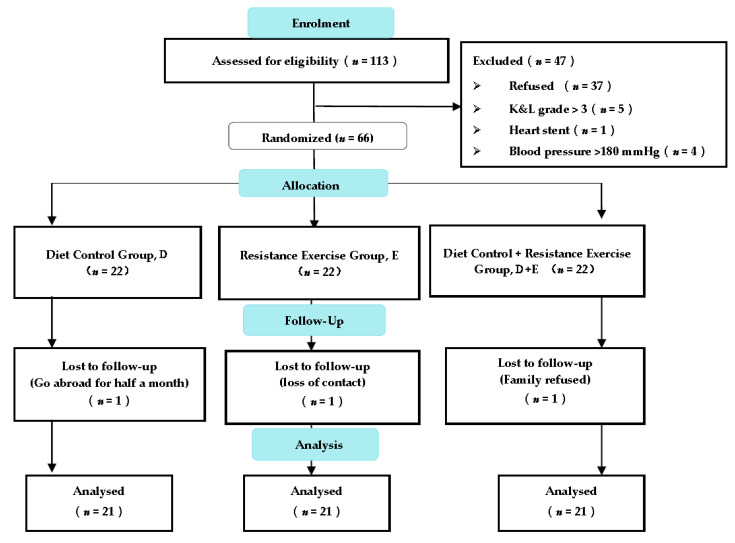
Consolidated Standards of Reporting Trials flow diagram.

A total of 113 outpatient screenings occurred during the enrolment period; 47 patients did not meet the inclusion criteria, and 66 patients were enrolled. Subsequently, the enrolled patients were assigned randomly to the D, E, or D + E groups. This study applied an intention-to-treat analysis. Of the 66 patients, 3 interventions were not completed due to an inability to conduct follow-up (overseas travel, loss of contact, family refusal). Ultimately, 63 participants (i.e., 21 in each group) completed the intervention (Figure 2). The adherence rates of the participants who completed the intervention were 83%, 90%, and 87% for the D, E, and D + E groups, respectively. No significant differences were observed among the three groups with respect to their baseline data (i.e., age, gender, height, weight, BMI, body fat percentage, K&L classification, and medication use; Table 1). Over the entire intervention period, no adverse events related to the intervention were reported.

### 3.2. Primary Outcomes

#### 3.2.1. Body Composition Analysis

Table 2 presents the postintervention changes in body composition. After the conclusion of the interventions, significant differences in body weight were observed among the three groups (F = 69.037, *p* < 0.001). In the pairwise comparison test, the D + E (*p* < 0.001) and D (*p* < 0.001) groups exhibited significant decreases in body weight relative to their baseline data. Furthermore, the changes in the D + E (*p* < 0.001) and D (*p* < 0.001) groups represented significant improvements relative to the E group. Significant differences in BMI were observed among the three groups (F = 66.788, *p* < 0.001). In the pairwise comparison test, the D + E (*p* < 0.001) and D (*p* < 0.001) groups exhibited significant improvements relative to their baseline data; however, such improvements were not observed in the E group (*p* = 0.161). Furthermore, the BMI changes in the D + E (*p* < 0.001) and D (*p* < 0.001) groups represented significant decreases relative to the E group. Significant differences in body fat percentage were observed among the three groups (F = 6.287, *p* = 0.003). In the pairwise comparison test, all groups (*p* < 0.001) exhibited significant improvements relative to their baseline data. Furthermore, the changes in body fat percentage in the D + E group represented significant improvements relative to the D (*p* = 0.019) and E (*p* = 0.012) groups. Significant differences in soft lean mass were observed among the three groups (F = 25.996, *p* < 0.001). In the pairwise comparison test, all groups exhibited significant improvements relative to their baseline data. Furthermore, the soft lean mass changes in the D + E (*p* < 0.001) and D (*p* < 0.001) groups represented significant improvements relative to the E group. Significant differences in lower-limb muscle mass were observed among the three groups (F = 43.255, *p* < 0.001). In the pairwise comparison test, a significant reduction and increase in lower-limb muscle mass were observed in the D and E groups, respectively, and no significant changes were observed in the D + E group (*p* = 0.178). Furthermore, the changes in the D group (*p* < 0.001) represented a significant reduction relative to the D + E group, and the changes in the E group represented a significant increase relative to the other two groups.

#### 3.2.2. WOMAC Analysis

Table 3 presents the postintervention changes in WOMAC scores. After the interventions concluded, significant differences in overall WOMAC scores were observed among the three groups (F =5.938, *p* = 0.004). In the pairwise comparison test, the D + E (*p* = 0.007) and D (*p* =0.017) groups exhibited significant improvements relative to their baseline data. Furthermore, the changes in the D + E group represented significant improvements relative to the D (*p* = 0.002) and E (*p* < 0.001) groups. The overall scores of the D, E, and D + E groups improved by 26.79%, 24.78%, and 33.52%, respectively. MCIDs were observed in all three groups.

For the pain subscale, no significant postintervention difference was observed among the groups (F =1.319, *p* = 0.275). However, all three groups (*P* < 0.001) exhibited significant improvements relative to their baseline data. Furthermore, the changes in the D + E group (*p* < 0.03) represented significant improvements relative to the E group. The pain scores of the D, E, and D + E groups improved by 32.16%, 29.64%, and 39.83%, respectively. MCIDs were observed in all three groups.

For the stiffness subscale, significant postintervention differences were observed among the groups (F =3.764, *p* = 0.029). In the pairwise comparison test, all three groups (*p* < 0.001) exhibited significant improvements relative to their baseline data. Furthermore, the changes in the D + E group (*p* = 0.001) represented significant improvements relative to the E group. The stiffness scores of the D, E, and D + E groups improved by 32.16%, 29.64%, and 39.83%, respectively. MCIDs were observed in all three groups.

For the physical function subscale, significant postintervention differences were observed among the groups (F =3.989, *p* = 0.024). In the pairwise comparison test, all three groups (*p* < 0.001) exhibited significant improvements relative to their baseline data. Furthermore, the changes in the D + E group represented significantly improvements relative to the D (*p* < 0.001) and E (*p* = 0.005) groups. The physical function scores of the D, E, and D + E groups improved by 23.05%, 21.54%, and 29.72%, respectively. MCIDs were observed in all three groups.

### 3.3. Secondary Outcomes

#### 3.3.1. Blood Biochemical Analysis

Table 4 presents the improvements in blood biochemistry values. For total cholesterol, significant postintervention differences were observed among the groups (F = 7.214, *p* = 0.002). In the pairwise comparison test, the D + E (*p* < 0.001) and D (*p* < 0.001) groups exhibited significant improvements relative to their baseline data; however, such improvements were not observed in the E group (*p* = 0.275). Furthermore, the changes in the D + E group (*p* = 0.001) represented significant improvements relative to the E group.

For LDL, significant postintervention differences were observed among the groups (F = 5.946, *p* = 0.004). In the pairwise comparison test, the D + E (*p* < 0.001) and D (*p* < 0.001) groups exhibited significant improvements relative to their baseline data; however, such improvements were not observed in the E group (*p* = 0.383). Furthermore, the changes in the D + E (*p* = 0.01) and D (*p* = 0.044) groups represented significant improvements relative to the E group.

For triglycerides, significant postintervention differences were observed among the groups (F = 6.035, *p* = 0.004). In the pairwise comparison test, the D + E (*p* < 0.001) and D (*p* < 0.001) groups exhibited significant improvements relative to their baseline data; however, such improvements were not observed in the E group (*p* = 0.325). Furthermore, the changes in the D + E group (*p* = 0.007) represented significant improvements relative to the E group.

#### 3.3.2. TUG Test

For the TUG test, significant postintervention differences were observed among the groups (F = 12.508, *p* < 0.001) (Table 5). In the pairwise comparison test, all groups (*p* < 0.001) exhibited significant improvements relative to their baseline data. MCIDs were observed in all groups. Furthermore, the changes in the D + E group (*p* = 0.001) represented significant improvements relative to the E group.

## 4. Discussion

To the best of our knowledge, this was the first prospective study in which patients with obesity and knee OA received home-based nutritional consultation combined with telemedicine-based exercise intervention. Individualized diet control interventions combined with telemedicine-based resistance exercise interventions, which were supervised by a clinical dietitian, significantly improved body composition (i.e., reduction in BMI and body fat percentage), improved blood biochemistry values (i.e., reduction in total cholesterol, LDL, and triglyceride levels), and enhanced lower-limb functional performance (i.e., improvements in WOMAC and TUG results) in the investigated population. The adherence rates of the patients who completed the intervention were 83%, 90%, and 87% in the D, E, and D + E groups, respectively. The high compliance can be attributed to the consistent follow-up with the clinical dietician and the convenience of exercising at home, which indicate that this method is feasible and effective. The adherence levels observed in all groups were higher than those reported in previous studies [16,46].

Regarding body composition changes, most weight-loss methods for patients with obesity and knee OA involve meal replacements. Although studies involving meal replacements have successfully achieved >10% body weight reductions, the rapid weight loss has also led to reductions in participants’ lean body mass by 11%–17% of their body weight [47,48,49]. In contrast, in the present study, each participant followed an individualized, balanced low-energy diet of 1200 kcal/day that was developed by a clinical dietitian. Each participant also engaged in follow-up and regularly consulted with the clinical dietitian through phone calls or online real-time communication once a week for 12 weeks; therefore, we were able to regularly monitor individuals’ nutritional status and control their energy intake such that their body weight could be reduced gradually. In addition, the percentage of participants who lost more than 5% of their body weight was 33.3%, and 47.6% in the E and D + E groups. A previous systematic review reported that a weekly reduction of body weight by 0.25% can improve the disability status of individuals with overweight or obesity with knee OA [50]. Moreover, we discovered that the participants in the D group experienced not only decreases in their body weight, body fat percentage, BMI, and soft lean mass, but also their muscle mass (which was expected). In the D + E group, no significant decrease in muscle mass was observed, which indicates that resistance exercise is essential for weight loss because it can slow muscle mass loss. In other words, implementing a weight-loss intervention that does not incorporate exercises will lead to the loss of muscle mass in the lower limbs and, consequently, increase the risk of knee joint impact injuries resulting from insufficient quadriceps muscle strength. Elastic band resistance exercises help to initiate muscle anabolism and improve the protein synthesis rate of the limb muscles. In the present study, soft lean mass and lower-limb muscle mass increased significantly in the E group. These results were consistent with those reported in a previous study [15]. Therefore, we recommend a combined telemedicine-based intervention for patients with obesity and knee OA because this intervention allows for weight loss without the loss of lower-limb muscle mass.

Regarding functional performance, the D + E group achieved the most significant improvements in overall WOMAC scores (33.52%) and physical function subscale (29.72%), and these improvements met the MCID threshold. Similarly to the present study, previous studies have indicated that elastic band resistance exercises improved the WOMAC scores of patients with and without obesity who have OA [13,51,52]. In addition, the D + E group exhibited significant improvements on the TUG test relative to the E group. This finding may have contributed to the decreased pain that was reported. The three groups experienced significant pain relief benefits after completing their interventions. Pain and physical activity are mutually influential [24]. A study of home-based balance and low-resistance exercise training verified that healthy older adults in the community who participated in a supervised exercise training program took an average of 8.7–9.91 s to complete the TUG test [53]. In the present study, after receiving telemedicine support from clinical staff and completing the interventions, the patients with obesity and knee OA took 9.37–9.92 s to complete the TUG test; these results are similar to those achieved by healthy older adults (8.4–9.1 s). This finding indicated a significant improvement that met the MCID threshold [42].

For blood biochemistry values, the three groups exhibited decreased total cholesterol levels. In addition, the D + E group exhibited lower LDL and triglyceride levels relative to the E group, indicating the effectiveness of regular diet control supervised by a dietician through telemedicine. Blood lipid level (particularly LDL level) influences a patient’s cardiovascular risk [54]. Previous studies have indicated that resistance exercise training aids the control of blood lipid levels [55,56]. Furthermore, a high triglyceride level increases endogenous LDL [57]. Collectively, these findings indicate that regular diet control and resistance exercise can greatly benefit patients at high risk of cardiovascular disease (e.g., those with obesity).

The primary contribution of the present study is its demonstration of the feasibility of implementing diet control through nutritional consultation and improving functional performance through a supervised resistance exercise program for the investigated population; this was achieved via phone consultations provided by clinical staff over a 12-week follow-up period after the participants’ first visit to the outpatient department. However, the study had several limitations that can be addressed in future studies. First, we enrolled participants from only a single medical center, and the positive outcomes could have been achieved primarily because a clinical dietitian with a sports medicine background aided in follow-up and tracked the health status and exercise adherence of the participants via phone calls or a communication application; in a regional hospital or small clinic, this protocol may be difficult to replicate. Second, other intervention-related dependent variables were not assessed. Changes in physical activity levels assessed through wearable technology (e.g., daily walking steps, speed), performance-based measures of physical function, and quality of life for this population were not tracked, and physical or occupational activities unrelated to the study could have introduced bias into the results. The OA participants were also not precisely assessed for intra-articular joint condition (e.g., cartilaginous wear) by magnetic resonance imaging [58] and biomechanics changes (e.g., gait, joint loading) [59]. Third, a potential placebo effect was not accounted for, since there is no sham exercise. Fourth, the severe OA participants (i.e., K&L grade >3) were excluded. The generalizability of the results to all patients with obesity and knee OA is low. Fifth, we did not verify whether the participants’ family members actively supervised the interventions, which is a factor that could also have introduced bias into the results. Sixth, limitations of BIA in morbidly obese patients may exist. Obese individuals have a relatively high amount of extracellular water and total body water, which may lead to underestimation of fat mass and overestimation of fat-free mass [60]. Therefore, adoption of total body DXA scanning for the precise measurement of body composition is suggested, but the safety of repeated measurements, the cost, and technical expertise are limiting [61]. A last, long-term follow-up should be conducted to determine, especially after the conclusion of interventions, whether our strategy changed the dieting and exercise behavior of the investigated population and produced long-term effects.

## 5. Conclusions

Supervised by a clinical dietitian, a 12-week individualized diet control intervention combined with a telemedicine-based elastic band resistance exercise intervention led to significant reductions in BMI, body fat, total cholesterol, LDL, and triglyceride levels and enhanced lower-limb functional performance in patients with obesity and mild-to-moderate knee OA. We recommend the application of this strategy by medical centers.

## Figures and Tables

**Table 1:** Baseline characteristics of participants.

Characteristics	D Group (*n* = 21)	E Group (*n* = 21)	D + E Group (*n* = 21)	*p*
Gender				
Male/female Age (year)	9/12 66.0 (3.9)	8/13 64.2 (4.1)	6/15 65.6 (3.9)	0.779 0.308
Body height (cm)	159.32 (5.64)	159.77 (7.44)	158.93 (6.82)	0.578
Body weight (kg)	78.37 (8.50)	79.00 (10.78)	77.53 (10.17)	0.891
Body mass index (kg/m^2^)	30.80 (2.58)	30.84 (2.47)	31.10 (2.60)	0.915
Body fat (%) waistline (cm)	35.18 (6.00) 102.12 (8.10)	34.64 (6.47) 102.07 (10.91)	36.2 (5.27) 100.35 (7.25)	0.690 0.764
Kellgren–Lawrence grade	1.76 (0.81)	1.62 (0.65)	1.81 (0.85)	0.528
I, *n* (%)	10 (47.6)	11 (50)	10 (47.6)	
II, *n* (%)	6 (28.6)	8 (38.1)	5 (23.8)	
III, *n* (%)	5 (23.8)	2 (9.1)	6 (28.6)	
Drug, *n* (%)				
Antihyperlipidemic	15 (71.4)	13 (61.9)	12 (57.1)	0.719
Antiarthritic	12 (57.1)	12 (57.1)	10 (47.6)	0.856
VAS (baseline)	6.19(1.03)	6.62 (1.17)	6.29 (1.06)	0.399

Values are means with standard deviations (in brackets) unless indicated otherwise. D: diet control group; E: resistance exercise group; D + E: diet control and resistance exercise group.

**Table 2:** Improvements in body composition after the 12-week intervention.

Outcomes	D	E	D + E
Body weight (kg)			
Baseline	78.37 ± 8.50	78.99 ± 10.78	77.53 ± 10.17
Post-intervention	74.96 ± 8.59	78.63 ± 11.00	74.03 ± 10.11
Change	−3.41 ± 0.75 ^$^	−0.36 ± 1.16	−3.50 ± 0.97 ^$^
Effect size	0.4	0.03	0.35
*p*	<0.001	0.168	<0.001
Body mass index (kg/m^2^)			
Baseline	30.8± 2.57	30.84± 2.47	31.10± 2.60
Post-intervention	29.45± 2.59	30.69± 2.59	29.7± 2.64
Change	−1.35 ± 0.33 ^$^	−0.15± 0.46	−1.41± 0.39 ^$^
Effect size	0.52	0.06	0.53
*p*	<0.001	0.161	<0.001
Body fat (%)			
Baseline	35.18 ± 6.01	34.64± 6.48	36.20± 5.27
Post-intervention	33.97 ± 6.47	33.48± 6.46	34.32± 5.50
Change	−1.20 ± 0.81	−1.17± 0.75	−1.87± 0.78 ^#,$^
Effect size	0.19	0.18	0.35
*p*	<0.001	<0.001	<0.001
Soft lean mass (kg)			
Baseline	46.29 ± 6.25	47.05 ± 8.41	45.64 ± 8.97
Post-intervention	45.05 ± 6.38	47.83 ± 8.53	44.46 ± 8.55
Change	−1.23 ± 0.65 ^$^	0.78 ± 0.64	−1.18 ± 1.56 ^$^
Effect size	0.2	0.1	0.13
*p*	<0.001	<0.001	0.002
Lower-limb muscle mass (kg)			
Baseline	17.59 ± 2.62	17.37 ± 3.2	17.34 ± 3.57
Post-intervention	16.85 ± 2.60	17.89 ± 3.37	17.17 ± 3.43
Change	−0.75 ± 0.40 ^$^	0.52 ± 0.35	−0.17 ± 0.55 ^#,$^
Effect size	0.28	0.16	0.05
*p*	<0.001	<0.001	<0.001

Results are expressed as means ± standard deviations. Change was calculated by subtracting the baseline value from the postintervention value; *p*: pairwise test; effect size indicates Cohen’s d; ^$^ indicates vs. E group (*p* < 0.05); ^#^ indicates vs. D group (*p* < 0.05); D: diet control group; E: resistance exercise group; D + E: diet control and resistance exercise group.

**Table 3:** Improvements in WOMAC scale after the 12-week intervention.

Outcomes	D	E	D + E
Total Score			
Baseline	34.57 ± 7.69	30.24 ± 6.06	38.86 ± 7.98
Post-intervention	25.33 ± 6.55	22.62 ± 5.41	25.81 ± 6.61
Change	−9.24 ± 3.59	−7.62 ± 2.64	−13.05 ± 4.09 ^#,$^
Effect size	1.29	1.33	1.78
*p*	<0.001	<0.001	<0.001
Pain			
Baseline	6.48 ± 2.21	6.05 ± 1.99	7.43 ± 2.01
Post-intervention	4.33 ± 1.65	4.14 ± 1.46	4.48 ± 1.44
Change	−2.14 ± 1.28	−1.90 ± 1.48	−2.95 ± 1.12 ^$^
Effect size	1.1	1.09	1.69
*p*	<0.001	<0.001	<0.001
Stiffness			
Baseline	2.91 ± 1.09	1.33 ± 1.15	2.86 ± 1.59
Post-intervention	1.86 ± 0.66	0.90 ± 0.83	1.38 ± 0.97
Change	−1.05 ± 0.81	−0.43 ± 0.68	−1.48 ± 1.03 ^$^
Effect size	1.17	0.21	1.12
*p*	<0.001	<0.001	<0.001
Physical Function			
Baseline	25.19 ± 5.62	22.86 ± 4.30	28.57 ± 5.76
Post-intervention	19.43 ± 5.29	17.76 ± 3.82	19.95 ± 4.79
Change	−5.76 ± 2.84	−5.10 ± 1.70	−8.62 ± 3.58 ^#,$^
Effect size	1.06	1.25	1.63
*p*	<0.001	<0.001	<0.001

Results are expressed as means ± standard deviations. Change is calculated by subtracting the baseline value from the postintervention value; *p*, pairwise test; Effect size indicates Cohen’s d; ^$^ indicates vs. E group (*p* < 0.05); ^#^ indicates vs. D group (*p* < 0.05); D, diet control group; E, resistance exercise group; D + E, diet control and resistance exercise group.

**Table 4:** Improvements in blood biochemistry values after the 12-week intervention.

Outcomes	D	E	D + E
Total cholesterol (mg/dL)			
Baseline	177.17 ± 23.35	169.95 ± 25.06	177.48 ± 27.04
Post-intervention	160.10 ± 20.16	165.81 ± 26.89	151.52 ± 18.03
Change	−17.57 ± 18.07	−4.14 ± 13.40	−25.95 ± 23.27 ^$^
Effect size	0.78	0.16	1.13
*p*	<0.001	0.175	<0.001
LDL (mg/dL)			
Baseline	99.47 ± 24.19	94.47 ± 24.19	97.57 ± 24.10
Post-intervention	84.38 ± 18.69	91.76 ± 24.32	79.52 ± 14.27
Change	−15.24 ± 15.17 ^$^	−2.71 ± 13.94	−18.05 ± 19.78 ^$^
Effect size	0.7	0.11	0.91
*p*	<0.001	0.325	<0.001
Triglycerides (mg/dL)			
Baseline	145.40 ± 52.95	128.81 ± 62.68	140.62 ± 54.57
Post-intervention	110.75 ± 48.77	118.05 ± 47.68	97.33 ± 35.79
Change	−34.65 ± 16.17	−10.76 ± 48.92	−43.29 ± 26.37 ^$^
Effect size	0.68	0.19	0.94
*p*	<0.001	0.325	<0.001

Results are expressed as means ± standard deviations. Change was calculated by subtracting the baseline value from the postintervention value; *p*: pairwise test; effect size indicates Cohen’s d; ^$^ indicates vs. E group (*p* < 0.05); D: diet control group; E: resistance exercise group; D + E: diet control and resistance exercise group.

**Table 5:** Improvements in TUG test after the 12-week intervention.

Outcomes	D	E	D + E
TUG(s)			
Baseline	11.25 ± 2.15	10.51 ± 1.97	10.98 ± 1.98
Post-intervention	9.92 ± 1.68	9.59 ± 1.56	9.37 ± 1.61
Change	−1.33 ± 0.59	−0.92 ± 0.67	−1.61 ± 0.52 ^$^
Effect size	0.69	0.52	0.89
*p*	<0.001	<0.001	< 0.001

Results are expressed as means ± standard deviations. Change was calculated by subtracting the baseline value from the postintervention value; *p*: pairwise test; effect size indicates Cohen’s d; ^$^ indicates vs. E group (*p* < 0.05); D: diet control group; E: resistance exercise group; D + E: diet control and resistance exercise group.

## Data Availability

Data are contained within the article.
